# Supplementary material for: The Genome of Bacillus velezensis SC60 Provides Evidence for Its Plant Probiotic Effects
Source: Microorganisms. 2022 Apr 1;10(4):767. doi: 10.3390/microorganisms10040767 (PMC9025316; doi:10.3390/microorganisms10040767)
Supplement: Supplementary file 1 [file microorganisms-10-00767-s001.zip › microorganisms-1641952-supplementary/microorganisms-1641952 - Suppl Figure S1.pdf]

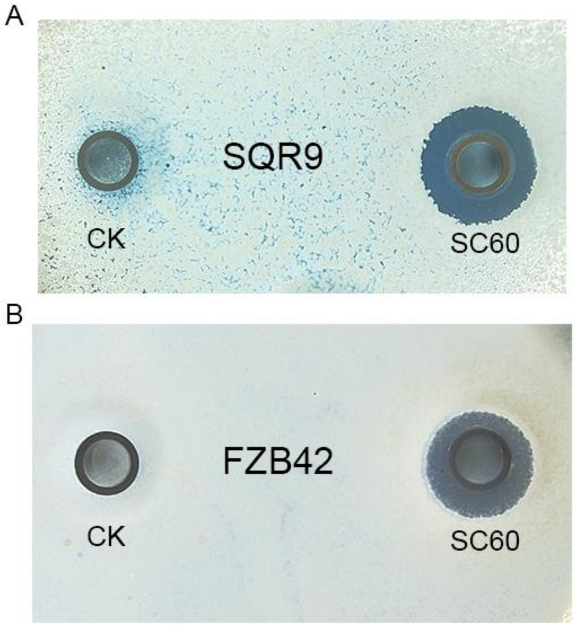

Figure S1. Antagonistic activity of strain SC60 against closely related *Bacillus velezensis*. A. Antagonistic activity of strain SC60 fermentation broth against *Bacillus velezensis* SQR9; B. Antagonistic activity of strain SC60 fermentation broth against *Bacillus velezensis* FZB42.
