# Supplementary material for: The Genome of Bacillus velezensis SC60 Provides Evidence for Its Plant Probiotic Effects
Source: Microorganisms. 2022 Apr 1;10(4):767. doi: 10.3390/microorganisms10040767 (PMC9025316; doi:10.3390/microorganisms10040767)
Supplement: Supplementary file 1 [file microorganisms-10-00767-s001.zip › microorganisms-1641952-supplementary/microorganisms-1641952 - Suppl Table S1.pdf]

Article

The Genome of *Bacillus velezensis* SC60 Provides Evidence for Its Plant Probiotic Effects

Xiaoyan Dong [1], Chen Tu [1], Zhihong Xie [2,*], Yongming Luo [1,3], Lei Zhang [1,4] and Zhaoyi Li [1]

1 CAS Key Laboratory of Coastal Environmental Processes and Ecological Remediation, Yantai Institute of Coastal Zone Research, Chinese Academy of Sciences, Yantai 264003, China; xydong@yic.ac.cn (X.D.); ctu@yic.ac.cn (C.T.); ymluo@yic.ac.cn (Y.L.); leizhang@iae.ac.cn (L.Z.); lizhaoyichloe@163.com (Z.L.)
2 National Engineering Research Center for Efficient Utilization of Soil and Fertilizer Resources, College of Resources and Environment of Shandong Agricultural University, Taian 271000, China
3 CAS Key Laboratory of Soil Environment and Pollution Remediation, Institute of Soil Science, Chinese Academy of Sciences, Nanjing 210008, China
4 Institute of Applied Ecology, Chinese Academy of Sciences, Shenyang 110016, China
* Correspondence: zhihongxie211@163.com

Table S1. Indicator strains used in this study.

Indicator strains	origin/reference
Bacillus velezensis	Laboratory stock
Bacillus halotolerans	Laboratory stock
Bacillus subtilis	Laboratory stock
Bacillus amyloliquefaciens	Laboratory stock
Bacillus fordii	Laboratory stock
Bacillus megaterium	Laboratory stock
Bacillus brevis	Laboratory stock
Bacillus cereus	Laboratory stock
Ralstonia solanacearum	Laboratory stock
*Fusarium oxysporum.*sp.*cucumebrium Owen*	Laboratory stock
Fusarium Verticillioides	Laboratory stock
Phytophthora capsici	Laboratory stock
Fusarium graminearum schw	Laboratory stock
Aspergillus niger Van Tiegh	Laboratory stock
Fusarium solani	Laboratory stock
Botrytis cinerea Pers.ex Fr	Laboratory stock
